# Feasibility and Effectiveness of Online Physical Activity Advice Based on a Personal Activity Monitor: Randomized Controlled Trial

**DOI:** 10.2196/jmir.1139

**Published:** 2009-07-29

**Authors:** Sander M Slootmaker, Mai J M Chinapaw, Albertine J Schuit, Jacob C Seidell, Willem Van Mechelen

**Affiliations:** ^3^Institute of Health ScienceFaculty of Earth and Life SciencesVU UniversityAmsterdamthe Netherlands; ^2^National Institute for Public Health and the EnvironmentBilthoventhe Netherlands; ^1^EMGO InstituteDepartment of Public and Occupational HealthBody@Work Research Center Physical ActivityWork and HealthTNO-VU University Medical CenterAmsterdamthe Netherlands

## Abstract

**Background:**

Inactive people are often not aware of the fact that they are insufficiently active. Providing insight into their actual physical activity (PA) levels may raise awareness and could, in combination with tailored PA advice, stimulate a physically active lifestyle.

**Objective:**

This study evaluated the feasibility and effectiveness of a 3-month intervention in which Dutch office workers were provided with a personal activity monitor (PAM) coupled to simple and concise Web-based tailored PA advice (PAM COACH).

**Method:**

Participants were randomly assigned to the 3-month PAM intervention (n = 51) or received a single written information brochure with brief general PA recommendations (n = 51). Study outcome measures were changes in PA (recall of minutes per week spent on PA, as measured by the Activity Questionnaire for Adolescents and Adults), determinants of PA, aerobic fitness, and body composition. Follow-up measurements were performed immediately after the 3-month intervention and at 8-months, 5 months after the end of the 3-month intervention period.

**Results:**

A total of 102 workers, 23 to 39 years old, completed the baseline measurement at the worksite. 48 completed the 3-month follow up and 38 the 8-month follow-up in the intervention group, 50 completed the 3-month follow up and 42 the 8-month follow up in the control group. 35 out of 48 (73%) participants in the PAM intervention group reported wearing the PAM regularly, and the PAM COACH was used almost once a week; 24 out of 46 (52%) PAM users set a personal goal, and 33 (72%) entered their favorite activities on the website. Main reasons for not using these items were lack of interest or not being able to find the item on the website. The majority of PAM users (34 out of 46, 74%) read the advice, of whom 14 (39%) found it unappealing. After the 3-month intervention, no significant intervention effect was observed (adjusted difference in min/week) for sedentary behavior (β = 10, 95% CI = −435 to 455), light-intensity PA (β = −129, 95% CI = −337 to 79), moderate-intensity PA (β = −13, 95% CI = −89 to 63), vigorous-intensity PA (β= −6, 95% CI = −75 to 62), and moderate- to vigorous-intensity PA (β = −23, 95% CI = −121 to 76). No significant intervention effect was observed in the PA outcomes at the 8-month follow-up. For the determinants of PA, aerobic fitness, and body composition, no statistically significant intervention effect was observed in the total study population immediately after the 3-month intervention or the 8-month follow-up.

**Conclusions:**

The intervention appeared to be easily applicable to real-life settings. The intervention was ineffective in improving PA behavior or its determinants in healthy office workers. More attention should have been given to the quality and appropriateness of the tailored advice.

**Trial Registration:**

International Standard Randomized Controlled Trial Number (ISRCTN): 93896459; http://www.controlled-trials.com/ISRCTN93896459/ (Archived by WebCite at http://www.webcitation.org/5iR3mf7ex)

## Introduction

According to the current Dutch Public Health physical activity (PA) recommendation, [1] adults should accumulate at least 30 minutes of moderate-intensity PA on at least 5 days of the week, or a minimum of 20 minutes of vigorous-intensity aerobic PA on 3 days of the week to promote and maintain health. Surveillance data from the Netherlands [2] and the United States [3] have shown consistent but low adherence (55% and 45%, respectively) to this recommendation among adults in general but have also shown large differences among subpopulations [4,5]. It is suggested that adults in full-time employment or those going through life events such as marriage and having children are more at risk of becoming physically inactive due to increased commitments [6].

Inactive subjects are often not aware of the fact that they are insufficiently active. This was recently shown in a survey of 2600 Dutch adults [7]. No less than 60% of the people who did not meet the recommendation believed that they were sufficiently active. The use of a PA monitor (eg, pedometer, accelerometer) that continuously registers and displays the actual PA level of the user may raise awareness and could thus overcome the problem of poor self-evaluation [8]. Hence, objective instant feedback by a PA monitor could positively affect the PA level in inactive subjects [8,9]. Hultquist et al [10] found that sedentary women who were given pedometers and who were instructed to walk 10,000 steps a day walked almost 2000 steps per day more than women who were instructed to go for a brisk 30-minute walk each day. PA monitors can be worn without major inconvenience, require little effort of the user, and are compatible with most daily activities, making them a practical and socially acceptable measure of PA [11].

Internet-based self-management interventions for PA have been shown to have potential [12-15] because they can reach large numbers of at-risk participants in a variety of settings at any time and location. There is evidence that health-related behavior is more affected by a tailored approach than by general health promotion activities [16,17]. Computer-tailored PA promotion programs are relatively new [18]. They provide respondents with individually adapted feedback about their current PA level and additionally provide individualized suggestions to change sedentary behavior and to promote daily PA. To date, little evidence is available on the feasibility and effectiveness of Internet-based tailored interventions coupled to an activity monitor [19,20].

The PAM concept (PAM BV, Doorwerth, the Netherlands) combines the use of a personal activity monitor (PAM) with simple and concise Web-based tailored PA advice (PAM COACH). The PAM (model AM101, PAM BV, Doorwerth, the Netherlands) is an uni-axial accelerometer in the vertical direction that can be easily attached to a belt. The validity of the PAM accelerometer has been tested in a laboratory setting and has shown results similar to the MTI Actigraph for estimating energy expenditure in walking and stair walking [21]. The PAM produces a single index score that accumulates during the day and is a proxy measure of total daily PA. The PAM shows the PAM score continuously on its display. Via a docking station, which must be connected to a computer with an Internet connection, the user can upload his or her personal PAM scores through PAM software to the PAM COACH website at any time throughout the day. On the PAM COACH website, users can interactively plan and evaluate their own activity advice based on their actual PAM scores and their PA goals and preferences.

The objective of this study was to evaluate the feasibility and effectiveness of providing a PAM coupled to simple and concise Web-based personalized PA advice on the daily PA level of young Dutch inactive office workers in a randomized controlled trial (RCT). In addition, the effects on determinants of PA, aerobic fitness, and body composition were examined. We hypothesized that the use of a PAM combined with individually tailored PA advice would increase awareness and subsequent PA levels of inactive office workers.

## Methods

### Study Design and Population

**Figure 1:**
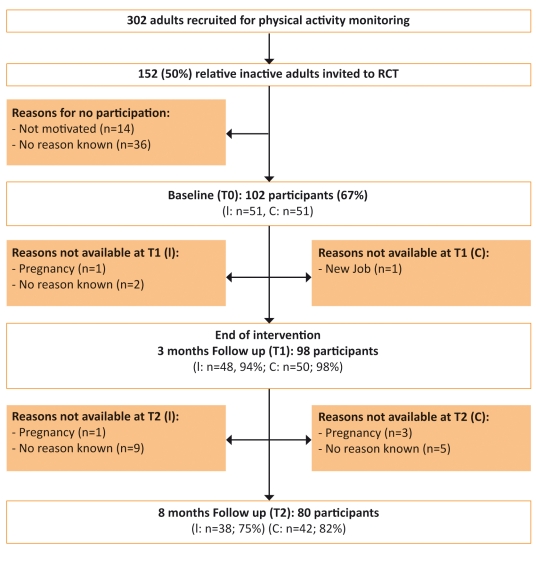
Flowchart of the intervention (I) and control (C) subjects in the RCT. Note that for analysis of the primary outcome measure at t1, 2 out of 48 (4%) and 1 out of 50 (2%) participants in the intervention and control group respectively, were excluded for analysis due to impossible values for physical activity (not shown in figure, see text under "Primary Outcome Measure").

This RCT is part of the PAM project, which is described extensively elsewhere [22]. Mainly office workers from 20 to 40 years old, all apparently healthy, with differing levels of education were recruited from eight worksites in the surrounding areas of Amsterdam, the Netherlands. For all worksites, the same recruitment protocol was used. In the recruitment procedure, we informed the participants about the beneficial health outcomes of regular PA (ie, increased cardiovascular health, and reduction of the risk of overweight, type 2 diabetes, depression, and some types of cancer) by providing a brochure and through individual communication. Inclusion criteria were ability to walk without aid, Dutch speaking, and not being pregnant. First, PA levels were monitored for 2 weeks by means of a PAM and a PA questionnaire. Based on these 2 weeks, the study population (N = 302) was divided into “active” (most active 50% of the population) and “inactive” (least active 50% of the population) groups. The inactive adults were invited to participate in the RCT and were told that they were eligible because of their low level of PA. To be able to detect a between-group difference of 20% in PA level (80% probability and a significance level of .05), two groups of 50 participants were required. Of the 152 invited adults, 102 (67% response rate) completed the baseline measurement and were randomly assigned to either the intervention (n = 51) or the control group (n = 51). Randomization to the intervention or control group was performed at the individual level by choosing sealed envelopes after the baseline measurements. The flow of subjects through the RCT and the distribution of nonresponders are shown in Figure 1. This study was approved by the Medical Ethics Committee of VU University Medical Center and was conducted between September 2004 and November 2005. All participants gave their informed consent.

### Intervention

After randomization, participants in both the intervention and control groups were advised to increase their PA levels. The control group received a single written information brochure with brief general PA recommendations. This print brochure is published by the Netherlands Heart Foundation and contains brief general information on the health benefits of PA and the PA recommendation. Everyone can obtain the brochure free of charge.

**Figure 2:**
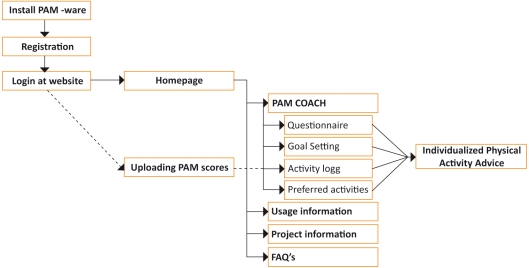
Functionalities of the PAM COACH website

 The intervention group received the PAM and was provided with Web-based tailored PA advice (PAM COACH) [23] for a 3-month period. Figure 2 and Table 1 show the sitemap of the PAM COACH website.

**Table 1:** Contents of the PAM COACH website

Website Section	Description
Home page	Presentation of the latest PAM week score, hyperlink to complete advice and motivational PA tips.
Goal setting	Setting the PAM goal score, indicated by the deficiency in minutes per day for their preferred activities.
Activity log	Presentation of all uploaded PAM scores (per day/week/month).
Questionnaire	Twelve questions on perceived physical activity barriers (yes or no).
Preferred physical activities	Categories: transport, school activities, in and around the house, individual and team sports.
Individualized PA advice	Translation of PAM goal score in the deficiency of minutes per day for their preferred physical activities. Feedback on their answers to the questionnaire. Stimulating feedback. Comparison of users’ PAM score to their peers in the intervention group.
Usage information	Information about the use of the PAM and PAM COACH website, including a demonstration.
Project information	The aim of the project and contact information.
FAQs	Answers to frequently asked questions about the (use of) PAM and PAM COACH website.

**Figure 3:**
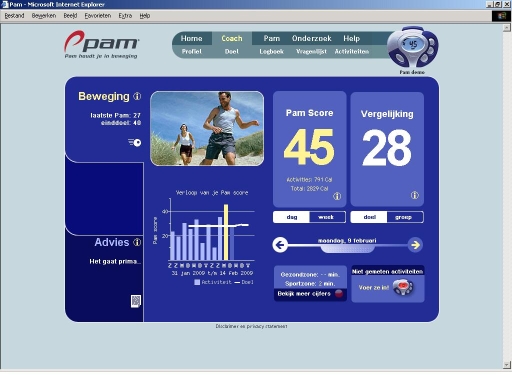
Screenshot of the PAM COACH website

First, the participants had to install the PAM software on their computer in order to use the PAM reader. When reading the PAM, the participant is automatically directed to the PAM COACH website. The user can register on the PAM COACH website by filling out a form with personal data (ie, username and password) and answering 12 questions on perceived PA barriers. Upon entry to the PAM COACH website, the user formulates a PAM goal score for the 3-month intervention period. Based on the user’s uploaded PAM score for the first week, the PAM COACH assigns a lower goal that increases daily until the PAM goal score is reached at the end of the intervention period. The PAM goal score can be changed by the user throughout the intervention. On every subsequent log-in, the PAM COACH website presents all the uploaded PAM scores and coupled PAM goals in orderly graphs per week or month (Figure 3).

The uploaded PAM scores are automatically accompanied by tailored PA advice on the computer screen as well as motivational tips for increasing PA (n = 21). The advice includes information on how to reach the PAM goal, which is based on (1) the user’s preferred activities (eg, an extra 60 minutes walking, or 25 minutes running, or 20 minutes playing squash daily), and (2) user-perceived PA barriers. Furthermore, the feedback is given in a stimulating way (eg, “You are doing better, but to reach your goal you have to do more”). If the PAM is not worn during certain activities (eg, swimming), these minutes can be added manually to the PAM score on the website. Apart from the short feedback on the PAM COACH website, the users can easily monitor their progress in daily PA by reading their PAM score directly on the display of the PAM. The participants received written and verbal instructions and practical demonstrations on how to wear the PAM and how to use the PAM COACH website (ie, setting a personal goal and favorite activities). Participants were instructed to register and upload PAM data in the first week of the intervention, to check if the system worked properly. After that, the participants could make use of the PAM and PAM COACH website as much as they wanted. At least one computer with PAM software and Internet access was available at all worksites except one, where ICT policies did not allow PAM software on the network. Participants from this worksite accessed the PAM COACH website at home only.

### Measurements

All measurements took place at the worksite during working hours, at baseline, immediately after the 3-month intervention, and at an 8-month follow-up, which corresponds to 5 months after the end of the 3-months intervention period. The measurements are described in detail elsewhere [22]. Gender, age, and education level were obtained at baseline. Education level was categorized into low and high. High education level comprised higher vocational education or a university degree. All other levels of education were defined as low.

#### Primary Outcome Measure

The Activity Questionnaire for Adolescents and Adults (AQuAA) was used to assess the amount of minutes per week spent on light-intensity (2-4 metabolic equivalents, METs), moderate-intensity (4-6.5 METs), and vigorous-intensity (> 6.5 METs) PA, as well as time spent sedentary (< 2 METs), such as watching TV and using the computer. The AQuAA refers to activities in the past week (7-day recall). Participants filled in the questionnaire while supervised by a research assistant. The research assistant checked the questionnaires when they were returned. Based on the assumption that one sleeps 8 hours per day, sixteen hours (960 minutes) was considered the maximum amount of time per day a person can spend on PA. At 3-months follow up, 2 out of 48 (4%) and 1 out of 50 (2%) participants in the intervention and control group respectively, were excluded because they exceeded this maximum time.

#### Secondary Outcome Measures

##### Determinants of Physical Activity

A short questionnaire was developed to assess behavioral intention to participate in sports more often. Intention to exercise was assessed by a single question: “Do you intend to participate more frequently in sports over the next three months?” For the determinants of attitude, social influences, and self-efficacy expectations and personal barriers toward sport (Cronbach alpha: .49, .85, and .78, respectively), a selection of two or three relevant questions was made, based on previous studies [24-27]. Answering formats were 5-point Likert scales (very low to very high). Per determinant, multiple items were converted into summary scores.

Awareness of complying with the Public Health PA recommendations was assessed by self-reported answers to the following questions: “On how many days of the week did you spent at least 30 minutes of moderate activity” and “Do you think you spend enough time participating in sports?” (yes or no). According to a method described by Ronda et al [7], respondents were allocated to four categories of awareness (underestimators, overestimators, realists adequate, or realists inadequate) based on their self-rated compliance with the PA recommendations and the results of the PA questionnaire. In the analyses, awareness was dichotomized in nonrealists (underestimators and overestimators) and realists (realists adequate and realists inadequate). Participants were classified as complying with the recommendation when they reported at least 150 minutes of moderate- to vigorous-intensity activity per week. Subjects’ knowledge of the PA recommendation was tested by the question “How much time per day do you have to spend on PA to stay healthy?”

##### Aerobic Fitness

A submaximal test, the Chester Step Test (CST) [28], was used to predict maximal aerobic capacity. The CST consists of five increasing paces of stepping on and off a bench. A step height of 30 cm was used for active participants and 20 cm for inactive participants. The CST starts at the relatively slow pace of 15 steps per minute and increases every 2 minutes to 20, 25, 30, and 35 steps per minute. Throughout the test, the heart rate (HR) is monitored. After each stage, the subject is asked to rate his or her perceived exertion on the Borg scale, which is a 15-point numerical rating scale ranging from 6 (very very light) to 20 (exhaustion). The test is terminated when the subject’s HR reaches 80% of the age-estimated maximal HR (ie, 220 minus age) or when the subject rates 14 on the Borg scale. Prior to this test, the subjects were screened with the Physical Activity Readiness Questionnaire (PAR-Q) [29]. Based on this screening, five subjects were excluded. Maximal aerobic capacity (VO_2_max) was predicted by the provided CST calculator (ASSIST Creative Resources Limited, Wrexham, England). This is based on the extrapolation of a line of best fit, which passes through the submaximal HR responses for each stepping stage up to a level that equals the participant’s age-estimated maximal HR.

##### Body Composition

Standard procedures were used to measure body weight, body height, waist and hip circumference, and the thickness of four skin folds (biceps, triceps, subscapular, and suprailiac). Body weight was measured in light clothing without shoes. Body mass index (BMI) was calculated by dividing the weight (kg) by height squared (m^2^). Percentage body fat was estimated from the sum of the four skin fold thickness measurements according to an age- and gender-specific method by Durnin and Womersley [30]. Before the baseline measurement, intrarater and interrater reliability for all four skin folds were determined. Intrarater reliability and interrater reliability (intraclass correlation coefficient, ICC) varied between .83 and .98.

#### Process Measures

After the 3-month intervention, PAM users were asked to evaluate (1) the PAM (ie, appreciation of the PAM score, frequency of wearing the PAM), (2) the PAM COACH website (ie, appreciation of the website, use of activity goal, and favorite activities), and (3) the tailored advice (ie, reading and appreciation of the advice). The uploaded PAM scores and the log-in frequency to the PAM COACH website were registered for each participant in the intervention group.

### Data Analysis

To compare baseline values, the chi-square test was used for gender, education, and awareness distributions. Nonparametric testing (Mann-Whitney U test) was used for PA data. Independent samples t-test was used to analyze all other demographic variables, determinants of PA, aerobic fitness, and body composition. The effect of the intervention was estimated based on the intention-to-treat principle, including all participants who had attended at least one follow-up measurement. Logistic regression analysis was used for the dichotomous outcome measure, awareness of meeting the PA, and sports recommendation (0 = nonrealists, 1 = realists). For all other outcome measures, standard linear regression analysis was used to test the differences between intervention and control groups at follow-up. The follow-up measurements were defined as dependent variables. Baseline values of the particular dependent variable were always included as covariates. The parameters of interest are the regression coefficients (β), indicating the effect of the intervention of interest compared to the control group. In a second analysis, gender (0 = male, 1 = female), age (continuous), education (0 = low, 1 = high), and BMI (continuous; not added in the analyses for body composition outcome measures) were considered as possible confounders or effect modifiers. If the interaction between group allocation and the variable concerned had a p-value below .10, then subgroup analyses were performed. Additionally, we adjusted for program adherence on the outcomes by performing regression analyses among intervention participants, including the log-in frequency. Analyses were performed using SPSS version 14.0 (SPSS Inc, Chicago, IL, USA).

## Results

### Study Population and Baseline Measurements

Table 2 shows baseline characteristics of both the intervention and control groups; 61 out of 102 participants were female (60%), and 66 out of 102 (65%) had a high level of education. Participants in the intervention and control groups were comparable except for age (intervention group was, on average, 1.3 years older; P = .05) and intent to participate in sports at baseline (higher in control group; P = .01). The majority of participants (68 out of 102, 67%) met the PA recommendation at baseline, with 78% (n = 32) of the men and 59% (n = 36) of the women meeting the recommendation.

**Table 2:** Baseline characteristics of total sample, PAM intervention group, and control group

Characteristic	Total (n = 102)	PAM (n = 51)	Control (n = 51)
**Demographics**			
Mean age in years ± SD	31.8 ± 3.5	32.5 ± 3.4	31.2 ± 3.5^a^
Female (%)	60	61	59
Highly educated (%)	65	63	67
Familiar with PA recommendations (%)	63	59	67
Compliance with PA recommendations (%)	67	69	65
**Determinants of participating in sports (mean ± SD)^b^**			
Attitude	4.25 ± 0.69	4.30 ± 0.75	4.25 ± 0.64
Self-efficacy	3.34 ± 0.71	3.22 ± 0.70	3.45 ± 0.71
Intention	3.44 ± 1.23	3.10 ± 1.20	3.78 ± 1.19^a^
**Awareness of compliance with recommendations (%)**			
Realist inadequate	23	23	23
Underestimator	27	27	27
Overestimator	10	11	9
Realist adequate	40	39	41
**Awareness of sports participation (%)**			
Realist inadequate	62	59	65
Underestimator	3	6	0
Overestimator	27	31	23
Realist adequate	8	4	12
**Aerobic fitness (mean ± SD)**			
VO_2_max (mL O_2_/kg/min)	41.4 ± 7.5	41.7 ± 8.4	41.2 ± 6.7
**Body composition (mean ± SD)**			
Weight (kg)	77.7 ± 14.6	79.0 ± 15.6	76.5 ± 13.6
BMI (kg/m^2^)	25.2 ± 4.1	25.9 ± 4.5	24.4 ± 3.5
Sum of skin folds (mm)	65.3 ± 31.6	69.4 ± 36.2	61.2 ± 25.8
Body fat (%)	27.1 ± 7.6	27.9 ± 8.0	26.4 ± 7.2
Waist circumference (cm)	85.4 ± 11.6	86.4 ± 11.9	84.5 ± 11.4

^a^Difference at baseline between intervention and control group (P< .05).

^b^Assessed on a 5-point Likert scale.

### Primary Outcome Measure

In the total study sample, our 3-month intervention did not significantly affect PA levels (Table 3). However, because of effect modification by education, we conducted subgroup analyses. These analyses showed that the 3-month intervention resulted in a relative lowering of light-intensity PA (2-4 METs) among higher-educated participants (adjusted difference between intervention and control group in min/week, β = −349, 95% CI = −632 to −66, P = .02). This effect was not sustained at the 8-month follow-up, 5 months after the intervention. A higher adherence to the program did not result in increased levels of PA (data not shown).

**Table 3:** Median PA scores and mean difference in PA and sedentary time between PAM intervention group and control group at baseline (n=51 in each group), 3 months (control: n=49; intervention n=46), and 8 months (control: n=42; intervention n=38)

Outcome Measure (min/week)	PAM	Control	Crude Difference^b^	Adjusted Difference^c^
	Median (IQR^a^)	Median (IQR^a^)	β (95% CI)	β (95% CI)
**Sedentary time**				
Baseline	3390 (2580; 3810)	3375 (2870; 3855)	–	–
3 months	3400 (2850; 3840)	2470 (2495; 3941)	101 (−338; 540)	10 (−435; 455)
8 months	2925 (2358; 4206)	3342 (2741; 3998)	−174 (−721; 374)	−267 (−803; 268)
**Light-intensity PA**				
Baseline	630 (480; 1320)	720 (450; 1220)	–	–
3 months	636 (345; 950)	678 (408; 1320)	−84 (−290.9; 123.3)	−129 (−337; 79)
8 months	500 (326; 994)	593 (323; 1020)	−18 (−220.6; 185.1)	−2.0 (−210; 206)
**Moderate-intensity PA**				
Baseline	90 (5; 240)	120 (10; 203)	–	–
3 months	75 (20; 180)	90 (8; 240)	−22 (−96; 53)	−13.0 (−89; 63)
8 months	120 (19; 241)	90 (8; 278)	97 (−47; 241)	103 (−42; 248)
**Vigorous-intensity PA**				
Baseline	170 (60; 315)	120 (30; 240)	–	–
3 months	80 (0; 210)	113 (41; 290)	−4 (−71; 63)	−6 (−75; 62)
8 months	120 (30;259)	115 (30; 303)	−17 (−97; 62)	−28 (−110; 54)
**Moderate- to vigorous-intensity PA**				
Baseline	320 (120; 510)	240 (75; 443)	–	–
3 months	197 (100; 480)	281 (150; 488)	−27 (−123; 68)	−23 (−121; 76)
8 months	223 (150; 548)	263 (143; 420)	81 (−109; 272)	74 (−119; 267)

^a^Interquartile range between 25th and 75th quartile.

^b^Baseline values of the particular dependent variable were always included as covariate.

^c^Adjusted for gender, age, education, and BMI at baseline.

### Secondary Outcome Measures

#### Determinants of Physical Activity

For the determinants of PA, no statistically significant intervention effect was observed in the total study sample (Table 4); however, an intervention effect was observed in subgroups of BMI. The proportion of subjects being aware of their adherence to the sports recommendation increased among overweight participants in the intervention group. The adjusted odds ratio (OR) between intervention (n = 16) and control group (n = 21) was 16.4 (95% CI = 1.3 to 214, P = .02). This significant effect was not sustained at the 5-month follow-up after the intervention.

#### Aerobic Fitness

No statistically significant intervention effect was observed on aerobic fitness.

#### Body Composition

No statistically significant intervention effect was observed on body composition in the total study population. However, subgroup analyses showed a decrease in body weight among low-educated intervention participants compared to their peers in the control group (adjusted difference, β = −1.6 kg, 95% CI = −2.8 to −0.4, P = .01). This difference was still observable at the 8-month follow-up, 5 months after the intervention (adjusted difference, β = −2.1 kg, 95% CI = −4.4 to 0.3, P = .08).

**Table 4:** Effectiveness of the 3-month PAM intervention on determinants of PA, aerobic fitness, and body composition: results of regression analyses

Outcome Measure	Crude Difference^a^	Adjusted Difference^b^
	β (95% CI)	β (95% CI)
**Determinants of playing sports (5-point Likert scale)**		
Attitude	−0.18 (−0.40; 0.04)	−0.20 (−0.43; 0.02)
Social influence	−0.01 (−0.31; 0.28)	−0.05 (−0.36; 0.24)
Self-efficacy	0.64 (−0.14; 0.27)	0.05 (−0.15; 0.26)
Intention	0.27 (−0.20; 0.74)	0.30 (−0.20; 0.80)
**Aerobic fitness**		
VO_2_max (mL O_2_/kg/min)	1.28 (−1.34; 3.90)	1.82 (−0.73; 4.39)
**Body composition**		
Weight (kg)	−0.27 (−1.12; 0.57)	−0.36 (−1.23; 0.49)
Sum of skin folds (mm)	1.49 (−4.38; 7.38)	1.34 (−4.62; 7.30)
Waist circumference (cm)	−0.51 (−1.85; 0.82)	−0.73 (−2.10; 0.63)
		
	OR (95% CI)	OR (95% CI)
**Awareness (%)^c^**		
Compliance with PA recommendations	1.45 (0.62; 3.37)	1.33 (0.54; 3.27)
Sports participation	0.81 (0.31; 2.11)	0.62 (0.22; 1.75)

^a^Baseline values of the particular dependent variable were always included as covariate.

^b^Adjusted for gender, age, education, and BMI at baseline. BMI at baseline was not added as confounder in the analyses for the body composition outcome measures.

^c^Awareness was analyzed with logistic regression (nonrealists = 0, realists = 1).

### Process Measures

Of the PAM users, 35 out of 48 (73%) reported to have worn the PAM “regularly” to “often” (Table 5). This finding was supported by the log-in frequency (almost once a week) of the PAM data to the PAM COACH website. Just over half of the PAM users (24 out of 46, 52%) set a personal goal, and 33 users (72%) entered their favorite activities on the website. Main reasons for not using these items were lack of interest and not being able to find them on the website. The tailored advice was read by 34 out of 46 (74%) PAM users, of whom 14 did not find the advice appealing. Main reasons were as follows: the advice was not personal or specific enough (n = 9), the advice was not applicable to their daily situation (n = 6), little variety in the advice (n = 3). Overall, the participants rated the PAM and the PAM COACH website as sufficient.

**Table 5:** Process evaluation data of the PAM accelerometer and the PAM COACH website

Variable	No.	Mean ± SD
**Log-in frequency to the PAM COACH website**		0.9 ± 0.6
1st month of intervention	47	3.8 ± 2.5
2nd month of intervention	47	3.6 ± 2.6
3rd month of intervention	47	3.4 ± 3.6
**Mean uploaded PAM score to the website**	26	
1st month of intervention		18.4 ± 7.8
2nd month of intervention		16.7 ± 7.5
3rd month of intervention		17.8 ± 7.6
Appreciation of PAM score^a^	47	6.4 ± 2.1
Appreciation of PAM COACH website^a^	47	6.5 ± 1.9
		
	No.	%^b^
**Wore the PAM accelerometer**	48	
Never		2
Hardly ever		10
Sometimes		15
Regularly		38
Often		35
Set personal PAM goal on website	46	52
Entered favorite activities on website	46	72
Read personalized advice on website	46	74
Found advice on website appealing	36	39

^a^On a scale of 1 (very negative) to 10 (very positive).

^b^Percentages are based on self-report.

## Discussion

This study investigated the feasibility and effectiveness of providing a PAM in combination with simple and concise tailored PA advice delivered through the Internet. The primary aim of the intervention was to improve daily PA, but we also examined effects on secondary outcomes such as determinants of PA, aerobic fitness, and body composition. According to the intention-to-treat analysis, the PAM intervention did not result in increased PA levels of young Dutch office workers, nor did it improve any of the secondary outcomes. These results may partly be due to the fact that only 39% (n = 14) of the users found their PA advice appealing.

The use of a personal website seemed to be applicable at every worksite with an Internet connection as well as being a suitable mode of conducting PA interventions among young employees. Yet, our intervention seemed ineffective at promoting PA in the total study population. This is in contrast to previous controlled interventions [10,19,20,31-33], which showed that a PA monitor helped sedentary participants to set goals and motivated them to increase their PA. However, these studies were not designed as RCTs and included mainly overweight participants or patients with type 2 diabetes.

Although our study was not designed for subgroup analyses, we conducted them after observing significant effect modification. Among low-educated intervention subjects, we observed a decrease in body weight of 1.6 kg. This effect is considerable after 3 months and is clinically relevant. Moreover, the proportion of realists increased among overweight subjects, which makes this concept of self-monitoring and Web-based feedback interesting for future research in these specific target groups.

### Limitations

The results of this study must be interpreted in light of its limitations. First, our primary outcome and some of our secondary outcomes were based on self-report and therefore prone to misreporting. However, since we looked at changes in PA behavior, at least the bias associated with systematic errors is cancelled out. Nevertheless, we compared the self-reported total PA data with the objective uploaded PAM data among participants of the intervention group (data not shown). The PAM data confirm the decline in total PA as assessed by the self-report (change in median min/week: −147) after the 3-month intervention.

Second, our control group is not a truly non-intervention group because they received an information leaflet on PA. However, we do not expect changes in PA by providing such brochures only. Furthermore, our findings may reflect ceiling effects associated with a relatively active sample at baseline. Although the study was aimed at inactive employees, 35 out of 51 (69%) participants in the PAM intervention group and 33 out of 51 (65%) participants in the control group already met the PA recommendation at baseline. Even though this information was based on self-report, it seems likely that the participants selected for the RCT were in general more active and health conscious than the general Dutch population. This is supported by the facts that the percentage of subjects who were acquainted with the PA recommendation was high in both groups, and the percentage of subjects who were aware (realists) of their compliance with the PA recommendation and their regular participation in sports was high in both groups. The fact that we did not collect information about the participants’ willingness to become physically active can be considered as a limitation. In addition, our study results are mainly applicable to people who are employed at a workplace that allows personal Internet use, which limits the generalizability of our study.

Finally, the practical advice given on the website was partly based on the objectively monitored PAM score. Accelerometers are insensitive to certain types of movements, in particular, nonambulatory physical activities with arm and or limb movements, such as cycling and weightlifting. This limitation of the accelerometer may have reduced the accuracy and relevance of the advice given by the PAM COACH website, particularly for subjects who cycle a lot, which is common in the Netherlands. Although activities that are not accurately measured by the PAM can be included manually on the PAM COACH website, a study has shown that recipients of negative or unexpected feedback responded by doubting the accuracy and credibility of the feedback information [34]. This phenomenon may have discouraged our participants from achieving their personal PA goal.

### Strengths

Strengths of the study are its design, the easy to implement intervention, and the low dropout rate. This RCT was set up as a short-term minimal intervention strategy in order to make it easily applicable in real-life settings. During the intervention, PAM users received short personalized PA advice together with supportive practical advice to reach their personal PA goal. In order to reach this PA goal, the activity preferences of the user were taken into account so that PA could be more easily implemented into their daily life. After registering, the user could decide when and how often to log in to the PAM COACH website. In spite of the minimal contact during the intervention between the researcher and the participant, adherence was moderate to high. PAM users logged in 10 times on average during the 3-month RCT, which is almost once a week and is comparable with frequencies of website log-ins from previous studies (range 0.7 to 1.5 times per week) [35,36]. Moreover, during the intervention we observed a low dropout, only 3 (6%) and 1 (2%) out of the 51 participants for the intervention and control group, respectively. The recurrent visits and low dropout during the intervention suggest that participants were interested in new information and were acquainted with the technology.

The appreciation of the intervention materials differed largely among participants in the intervention group; most participants expected more varied and concrete advice and found the advice not applicable to their daily life. This occurred in spite of our aim to tailor the PA advice to a certain extent based on the users’ actual PAM score in relation to their PAM goal. We strived for simple and concise PA advice and a variety of motivational tips on the PAM COACH website.

### Conclusions

To conclude, we hypothesized that the combination of wearing a PAM combined with tailored PA advice delivered through the Internet would be potentially successful in increasing awareness of personal activity levels and actual PA levels. However, we did not observe any significant effect on awareness, PA level, determinants of PA, aerobic fitness, or body composition among the total group of young healthy Dutch employees. This may be explained by the fact that we conducted a minimal intervention in a study population that largely (67%) met the PA recommendations at baseline. Moreover, a large part of the intervention population did not find the advice appealing. Hence, the results of the present study do not give cause for wider implementation of this minimal intervention among healthy adults. Since we observed a tendency for a positive intervention effect on body weight among low-educated adults, more research may be necessary to investigate the effectiveness of this type of intervention among people who are overweight or of low socioeconomic status. In this, attention should be given to the quality and appropriateness of the tailored advice.

## Abbreviations

AQuAA: Activity Questionnaire for Adolescents and Adults
BMI: body mass index
CST: Chester Step Test
HR: heart rate
ICT: information and communication technologies
MET: metabolic equivalent
PA: physical activity
PAM: personal activity monitor
RCT: randomized controlled trial
